# Wearable devices in atrial fibrillation: screening, monitoring, and management

**DOI:** 10.3389/fcvm.2026.1887788

**Published:** 2026-07-21

**Authors:** Wen Chen, Xinyi Lin, Ping Jia, Zhenwei Liu

**Affiliations:** 1 University of Electronic Science and Technology of China, Chengdu, China; 2 Mianyang Third People’s Hospital, Mianyang, China; 3 Sichuan Provincial People’s Hospital, University of Electronic Science and Technology of China, Chengdu, China

**Keywords:** atrial fibrillation, digital health, electrocardiography, photoplethysmography, remote monitoring, screening, wearable devices

## Abstract

Atrial fibrillation (AF) remains the most prevalent cardiac arrhythmia worldwide, contributing substantially to the risk of ischemic stroke and heart failure. Brief monitoring windows inherently limit traditional episodic screening strategies and frequently fail to detect paroxysmal or asymptomatic occurrences. Wearable devices integrating multimodal sensors—such as photoplethysmography (PPG) and single-lead electrocardiography (ECG)—with artificial intelligence now enable continuous, real-world AF screening. However, the widespread consumer adoption of these technologies has precipitated a novel clinical dilemma. A marked increase in the detection of subclinical atrial fibrillation (SCAF) introduces considerable uncertainty regarding the appropriate threshold for initiating oral anticoagulation. In this review, we evaluate the underlying technical principles of wearable sensors and examine their integration into clinical practice across community screening, postoperative monitoring, and long-term patient management. We critically analyze emerging clinical challenges—focusing on the detection-vs.-intervention paradox emphasized by recent landmark outcome trials—and propose actionable strategies for future implementation. Successful clinical translation will require standardized computational algorithms, validated hardware accuracy, semantic electronic health record (EHR) interoperability, and the continuous quantification of AF burden to tailor personalized antithrombotic therapy.

## 1 Introduction

Atrial fibrillation (AF) is the most prevalent cardiac arrhythmia, currently affecting an estimated 59.7 million individuals globally. According to the 2023 ACC/AHA and 2024 ESC guidelines, AF increases mortality risk by 1.5- to 1.9-fold and accounts for approximately one-third of all ischemic strokes (1, 2). Regional epidemiological data mirror this global burden; the adult prevalence of AF in China is estimated at 1.6%, accompanied by a hospitalization rate of 43.7 per 100 person-years (3, 4). Early identification, accurate diagnosis, and timely intervention remain essential to mitigate these cardiovascular risks.

The paroxysmal and frequently asymptomatic nature of AF complicates early detection. Approximately one-third of all AF cases remain subclinical, contributing to persistently low detection rates with traditional, episodic electrocardiogram (ECG) assessments (5, 6). Commercial and medical-grade wearables offer a compelling solution. By coupling continuous physiological monitoring with artificial intelligence (AI), these devices automate AF identification and facilitate remote patient-clinician interactions via secure cloud platforms.

A rapidly expanding body of research explores the clinical utility of wearables across population screening, postoperative rhythm monitoring, and longitudinal home management. This review synthesizes current evidence regarding the engineering principles, clinical applications, and technological advancements of wearable devices in AF care. We also analyze the barriers hindering their widespread adoption and propose a strategic roadmap to accelerate their integration into clinical workflows.

## 2 Sensor modalities and intelligent signal processing in wearable devices

Contemporary wearable devices seamlessly integrate biosensors, signal acquisition modules, and data communication components into ubiquitous form factors, including smartwatches, chest straps, smart textiles, rings, and optical glasses (7). Electrodes capture direct bioelectrical activity, as seen in electrocardiography (ECG), whereas optical sensors, particularly photoplethysmography (PPG) probes, translate dynamic microvascular volumetric changes into interpretable electronic signals. The analytical processing of these raw signals has transitioned from conventional impulse response filters to dynamic adaptive filtering. The field increasingly incorporates advanced computational methodologies, including wavelet analysis, sophisticated pattern recognition, and self-learning artificial neural networks (7). These advancements drive continuous, high-precision physiological signal analysis in ambulatory settings.

### 2.1 Review methodology

To synthesize the landscape of wearable cardiovascular technologies, we conducted a comprehensive literature search across PubMed, Web of Science, Scopus, the China National Knowledge Infrastructure (CNKI), and Wanfang Data for articles published between January 2010 and March 2026. We restricted the search to English- and Chinese-language publications using combinations of key terms: “atrial fibrillation,” “wearable devices,” “photoplethysmography,” “electrocardiography,” “digital health,” “screening,” “remote monitoring,” and “subclinical atrial fibrillation.” We systematically queried landmark clinical outcome trials (e.g., NOAH-AFNET 6, ARTESiA), major society guidelines (including ESC, AHA/ACC, and Chinese guidelines), and high-impact validation cohorts, followed by a manual screening of reference lists to capture additional publications.

Given the substantial heterogeneity across device technologies, study designs, and target populations, we adopted a qualitative integrative framework rather than a formal systematic review or meta-analysis. Selection prioritized clinical relevance, methodological rigor, cohort size, and direct applicability to wearable-based AF management. To ensure scientific rigor and minimize selection bias, inclusion required: (1) original research or clinical trials evaluating wearable-based AF screening, monitoring, or management; (2) utilization of PPG or ECG modalities; and (3) clear diagnostic accuracy or quantitative clinical outcome data. Exclusion criteria comprised: (1) studies focusing solely on healthy subjects without arrhythmia assessment; (2) conference abstracts or posters lacking full-text peer review; and (3) retracted or duplicated publications. To ensure global relevance and mitigate regional bias, we systematically prioritized landmark international trials and major society guidelines.

### 2.2 Mechanisms of signal acquisition: ECG vs. PPG

Wearable technologies predominantly rely on two modalities for cardiac monitoring: ECG and PPG (7).

#### 2.2.1 ECG

Single-lead wearable ECG platforms—such as smartwatches equipped with titanium electrodes or adhesive chest patches—directly capture myocardial electrical activity. AF diagnosis relies on strict morphological criteria: the absence of distinct P waves (reflecting the loss of synchronized atrial depolarization), an undulating fibrillatory baseline, and absolute irregularity of R-R intervals (1). By providing direct visualization of atrial electrical activity with clear morphological features, ECG remains the clinical gold standard for AF identification.

#### 2.2.2 PPG

Rather than measuring bioelectrical activity directly, PPG utilizes optical sensors—typically green or infrared light-emitting diodes coupled with photodetectors—to capture photometric changes in cutaneous microvascular blood volume during each cardiac cycle (7, 8). AF manifests in PPG signals as irregular peak-to-peak pulse intervals alongside significant variations in pulse amplitude (7). A critical diagnostic limitation, however, is PPG's inherent inability to capture P-wave morphology. This deficit profoundly limits the capacity of basic PPG devices to distinguish true AF from mimicking arrhythmias, such as premature atrial or ventricular contractions (9).

### 2.3 Signal processing and clinical diagnostic challenges

Raw physiological signals acquired in ambulatory settings are inherently susceptible to noise; consequently, conventional peak-detection algorithms frequently generate substantial false-positive rates (10). In clinical practice, false-positive alerts from consumer-grade devices provoke patient anxiety and trigger cascades of unnecessary evaluations, including specialist consultations and supplementary testing (11). Bridging the translational gap between consumer electronics and clinical-grade diagnostic tools increasingly relies on artificial intelligence (AI) to optimize diagnostic specificity (12, 13).

Moving beyond simple irregular-pulse detection, contemporary machine learning models analyze complex R-R interval sequences and continuous heart rate variability metrics (13). This analytical depth enables algorithms to differentiate the chaotic rhythm of true AF from benign arrhythmias or ectopy. Nevertheless, false positives remain a formidable clinical concern in real-world environments (9).

Mitigating motion artifacts is equally critical for reliable continuous monitoring. Advanced AI architectures integrate concurrent kinematic data from triaxial accelerometers to identify and discard periods of excessive physical exertion. By restricting rhythm analysis exclusively to intervals of high signal fidelity, this multimodal approach minimizes the clinical burden of false alerts and prevents data overload for healthcare providers (14, 15).

### 2.4 Advanced algorithms, multi-sensor fusion and emerging sensing modalities

Advances in computational signal processing substantially augment the diagnostic utility of both consumer and medical-grade wearables. Two technological paradigms drive this progress: deep learning architectures for complex arrhythmia analysis, and multi-sensor fusion to mitigate environmental interference. Concurrently, novel non-contact sensing modalities offer a promising complementary approach.

#### 2.4.1 Deep learning architectures

Conventional machine learning algorithms rely on manually engineered biomarkers—such as R-R interval variability and isolated P-wave morphology—which frequently fail to capture subtle arrhythmic patterns embedded within noisy ambulatory signals. Deep learning models circumvent this limitation by automatically extracting hierarchical, discriminative features directly from unprocessed waveforms. Convolutional neural networks (CNNs) demonstrate considerable efficacy in mining spatial-morphological features from single-lead ECG data, thereby facilitating robust AF identification in large-scale ambulatory cohorts (16). To complement this spatial analysis, long short-term memory (LSTM) recurrent neural networks are optimized to model sequential temporal dynamics within R-R intervals over extended monitoring periods (17). Recently, transformer architectures utilizing self-attention mechanisms have emerged in continuous ECG analysis, offering enhanced capacity to capture subtle, long-range rhythm variations (18). While CNNs and LSTMs have undergone extensive validation, transformer-based models demand substantial training data and computational resources; their real-world generalizability across resource-constrained platforms remains under active investigation (19).

#### 2.4.2 Multi-sensor fusion

Single-mode wearable sensors are inherently susceptible to motion artifacts and ambient noise, frequently triggering false-positive alerts that erode clinical confidence. Contemporary hardware addresses this vulnerability by integrating complementary sensor arrays. State-of-the-art AI frameworks cross-reference optical PPG signals with concurrent kinematic data from three-axis accelerometers and gyroscopes. This integration enables dynamic adaptive filtering to eliminate low-fidelity signal segments and isolate artifact interference (7). Fusing multimodal data—pulse waveforms, movement intensity, and autonomic indicators—enhances the algorithm's capacity to differentiate genuine AF from benign sinus arrhythmia. This multidimensional approach specifically curbs false-positive alerts triggered by premature atrial or ventricular contractions (PACs/PVCs), which are prevalent in older adults and often mimic AF during basic pulse interval analysis (9). Multimodal smart rings exemplify this trajectory by incorporating PPG, thermistors, and accelerometers for comprehensive tracking, though clinically validated AF detection algorithms for these specific form factors remain under active development (19).

#### 2.4.3 Emerging sensing modalities

Non-contact radar monitoring represents a rapidly evolving frontier. Ultra-wideband (UWB) continuous-wave radar detects sub-millimeter micro-displacements of the chest wall induced by the cardiac cycle. This supports passive, longitudinal rhythm monitoring without requiring active patient compliance or physical hardware attachment—an ideal solution for elderly or multi-morbid patients who struggle with smartwatch or patch adherence. Despite this physiological rationale, current radar systems lack the large-scale, AF-specific validation necessary for definitive diagnosis, serving primarily as adjunctive tools for hemodynamic trend tracking. Preliminary studies support the feasibility of UWB radar for contact-free heart rate monitoring. Still, these investigations feature small sample sizes and lower overall diagnostic accuracy compared to established ECG modalities (19).

### 2.5 Comparison of wearable technologies

As emphasized in recent comprehensive evaluations of remote arrhythmic monitoring, such as the analysis by Tetaj et al. (19) encompassing populations with inherited cardiomyopathy and heart failure, wearable platforms exhibit substantial variability in sensor modalities, signal fidelity, and clinical applicability. Although continuous, AI-enabled monitoring was initially pioneered in high-risk cardiomyopathy cohorts to capture transient electrical instability and dynamic ectopic burden, these same technological platforms are now occupying an increasingly central role in AF screening and long-term management. To establish a structured technological framework for the subsequent clinical discussion, Table 1 delineates the core sensor modalities, measured physiological signals, optimal monitoring durations, and inherent advantages and limitations of the primary wearable technologies currently deployed across both clinical and consumer domains.

**Table 1:** Comparison of wearable technologies for atrial fibrillation screening and monitoring.

Wearable category	Sensor modality & measured signal	Monitoring duration	Clinical applications	Advantages	Limitations	Representative devices/studies
Smartwatch (PPG-based)	Photoplethysmography (PPG) detects blood volume changes (HR, HRV)	Continuous/Long-term	Passive AF screening in community populations	High user compliance enables continuous background screening at scale	Susceptible to motion artifacts and skin tone variations; lower specificity for AF vs. PACs/PVCs; lacks P-wave morphology; requires ECG confirmation	Apple Watch [Apple Heart Study (20)], Fitbit [Fitbit Heart Study (22)], Huawei Devices [Huawei Heart Study (21)]
Smartwatch (ECG-enabled)	Single-lead ECG via titanium electrodes; records electrical activity	Intermittent (typically 30-s recordings)	AF diagnosis confirmation; symptom-triggered monitoring	Provides an actionable rhythm strip for clinical review; high diagnostic specificity for AF when P-wave absence and RR irregularity are confirmed	Requires active user initiation; not suitable for continuous background rhythm monitoring	Apple Watch ECG feature (20)
Handheld ECG devices	Single or multi-lead ECG via finger pads	Intermittent	Symptom-triggered AF detection in high-risk patients	Clinical-grade signal quality; highly portable and easy to use	Episodic in nature, it heavily relies on patient symptom awareness and compliance	AliveCor KardiaMobile [REHEARSE-AF Study (25)]
Wearable ECG patches	Adhesive single-lead or P-wave-centric electrodes	Continuous (1–30 days, device-dependent)	AF detection, POAF monitoring, high-risk screening	High diagnostic yield; provides clinical-grade continuous data without requiring active user input	Requires prescription; potential for skin irritation/dermatitis; finite battery/monitoring duration	Zio Patch (27), Preventice BodyGuardian (26), mSToPS trial patch (26)
Smart rings	PPG, temperature, accelerometer	Continuous	Autonomic tracking, suspected AF alerts, and sleep monitoring	Extremely unobtrusive; excellent battery life and sleep-stage tracking capabilities	Relies entirely on peripheral perfusion; cannot provide ECG tracings for formal diagnosis; limited AF-specific validation data	Oura Ring (19)
Smart textiles/garments	Embedded textile electrodes capture ECG and respiration	Intermittent to continuous	Long-term ECG monitoring during daily activities	Integrates seamlessly into daily clothing; enables multimodal physiological sensing	Higher cost; requires washing maintenance; signal noise can increase during vigorous movement; limited large-scale validation data compared to smartwatch-based PPG and ECG patches; no published AF-specific validation studies	OmSignal system (28), Hexoskin (19)
Contact-free radar sensors	Ultra-wideband (UWB) continuous-wave radar; detects sub-millimeter chest wall micro-displacements	Continuous	Passive, non-contact rhythm monitoring; elderly/long-term care	Zero user burden; no skin contact required; suitable for multi-comorbid patients with poor adherence	Limited AF-specific validation data; lower signal fidelity compared to ECG; environmental interference may affect accuracy	Research-stage UWB radar systems (19)

AF, atrial fibrillation; ECG, electrocardiography; HR, heart rate; HRV, heart rate variability; PAC, premature atrial contraction; POAF, postoperative atrial fibrillation; PPG, photoplethysmography; PVC, premature ventricular contraction.

## 3 Current applications in AF care

**Figure 1:**
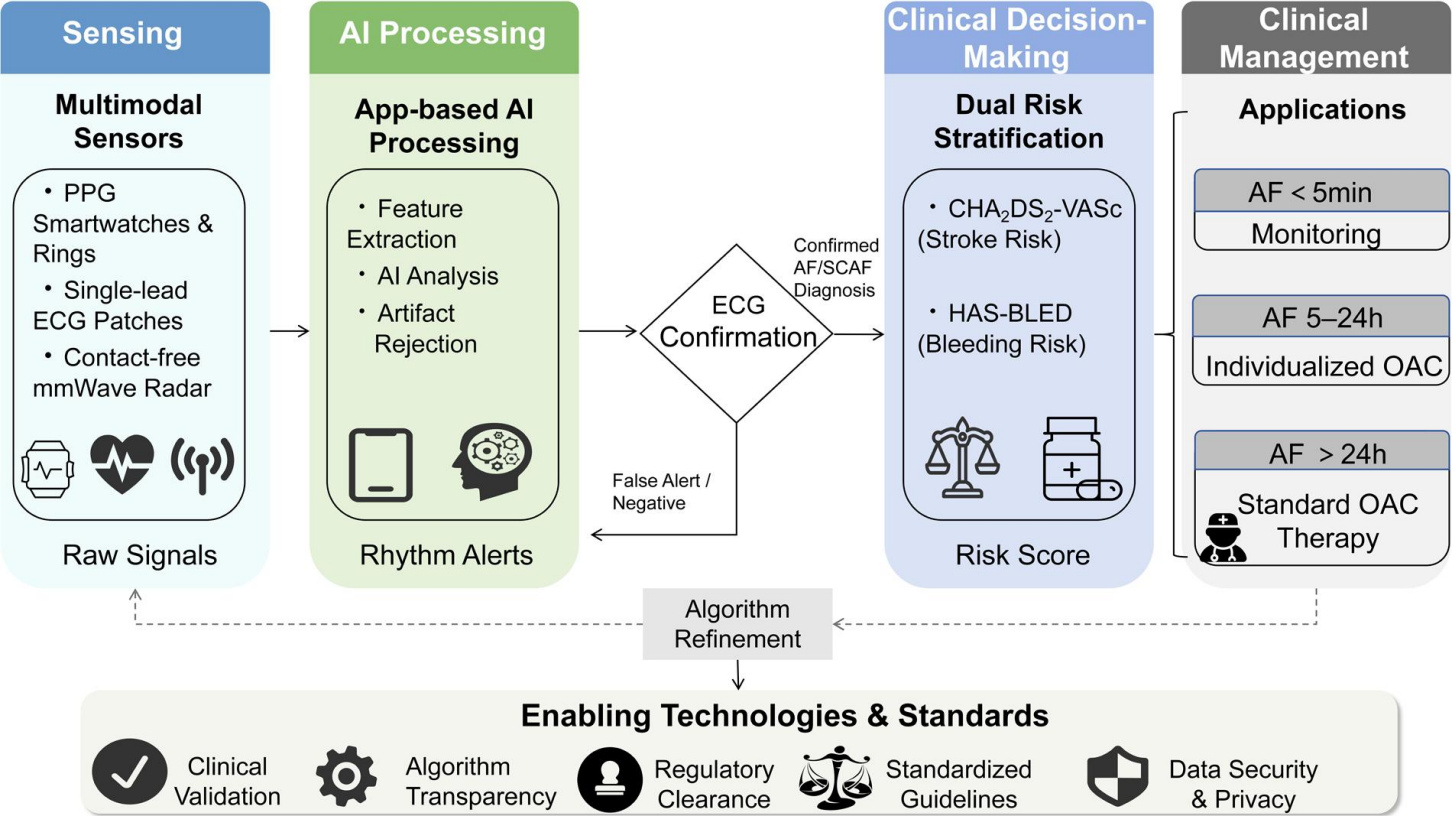
The closed-loop paradigm of wearable devices in atrial fibrillation (AF) management. Raw physiological signals acquired via multimodal sensors (PPG and single-lead ECG) are transmitted to smartphone-based applications. Utilizing feature extraction and artifact rejection algorithms, these applications generate real-time rhythm alerts. PPG-derived alerts serve only as preliminary screening results and must undergo ECG confirmation. False alerts return to continuous wearable monitoring, while confirmed AF/SCAF receives dual risk evaluation with CHA_2_DS_2_-VASc and HAS-BLED scores. Anticoagulation therapy is individualized according to AF episode duration. The whole workflow is supported by clinical validation, algorithm transparency, regulatory clearance, standardized guidelines, and data security and privacy. Clinical outcomes feed back to realize continuous algorithm refinement and form a complete closed-loop system. AF, atrial fibrillation; CHA_2_DS_2_-VASc, congestive heart failure, hypertension, age ≥75 years, diabetes, stroke/TIA, vascular disease, age 65–74 years, sex category; ECG, electrocardiography; HAS-BLED, hypertension, abnormal renal/liver function, stroke, bleeding history or predisposition, labile INR, elderly, drugs/alcohol concomitantly; PPG, photoplethysmography; SCAF, subclinical atrial fibrillation.

As illustrated in Figure 1, the integration of multimodal wearable sensors and app-based artificial intelligence forms a self-improving closed-loop digital health system. Starting with raw physiological signal acquisition, the system generates rhythm alerts via AI processing, followed by ECG verification, CHA_2_DS_2_-VASc/HAS-BLED dual risk evaluation, and individualized antithrombotic treatment. Clinical outcome data are then used to refine the algorithm iteratively. Based on this full technical pathway, we elaborate on its real-world implementation in three key settings: population-level community screening, postoperative surveillance, and long-term home-based care.

### 3.1 Screening in community and high-risk populations

Early AF screening anchors proactive cardiovascular risk management. PPG algorithms have been widely adopted for initial AF screening in consumer smartwatches (7, 8). The landmark Apple Heart Study demonstrated the feasibility of large-scale screening across 419,297 participants (20). Subsequent massive cohorts—including the Huawei and Fitbit Heart Studies (evaluating 187,912 and 455,699 individuals, respectively)—reported high positive predictive values (91.6% and 98.2%) for PPG-derived alerts upon standard ECG confirmation (21, 22) (Table 2).

**Table 2:** Landmark large-scale real-world studies on wearable devices for atrial fibrillation screening.

Study	Device modality & monitoring strategy	Study design	Sample size (*N*)	Population/mean age	Confirmation method	Key findings	Clinical context
Apple heart study (20)	Apple Watch (PPG); passive background monitoring	Prospective, single-arm	419,297	General population/∼41 years	ECG patch sent to notified participants	0.52% received irregular pulse notification; PPV 84% for AF on concurrent ECG patch; AF confirmed in 34% of notified participants who completed follow-up	Community screening
Huawei heart study (21)	Huawei smart devices (PPG); continuous passive monitoring	Prospective, single-arm	187,912	General population/∼35 years	Clinical evaluation + 12-lead ECG/Holter	0.23% received “suspected AF” notification; 87% of followed participants clinically confirmed AF; PPV 91.6%	Community screening
Fitbit heart study (22)	Fitbit wearables (PPG); background rhythm tracking	Prospective, single-arm	455,699	General population/∼47 years	1-week ECG patch	1.0% received irregular rhythm notification; PPV 98.2% for AF on concurrent ECG patch	Community screening
REHEARSE-AF (25)	AliveCor KardiaMobile (handheld single-lead ECG); twice-weekly + symptom-triggered	Randomized controlled trial (RCT)	1,001 (500 intervention vs. 501 control)	Age ≥65 years, CHA_2_DS_2_-VASc ≥2/72.6 years	Single-lead ECG over-read by electrophysiologists	AF detected in 3.8% (intervention) vs. 1.0% (control) at 12 months; HR 3.9 (95% CI 1.4–10.4); *p* = 0.007	High-risk screening
mSToPS (26)	Zio Patch; up to 4 weeks continuous monitoring, repeated at 4 months	RCT + prospective matched observational cohort	2,659 (1,366 immediate vs. 1,293 delayed)	High-risk (CHA_2_DS_2_-VASc risk factors), no AF history/72.4 years	Patch ECG analyzed by independent technicians	Primary endpoint at 4 months: AF newly diagnosed in 3.9% (immediate) vs. 0.9% (delayed); absolute difference 3.0% (95% CI 1.8%–4.1%)	High-risk screening
BASEL wearable study (36)	Apple Watch, Samsung, Fitbit, Withings, AliveCor (PPG + ECG); simultaneous testing	Prospective validation study	201	Patients with AHRE or known AF/67 years (median)	12-lead ECG	PPG sensitivity: Apple 89%, Samsung 85%, Fitbit 82%, Withings 79%; single-lead ECG diagnostic accuracy >95%	Device validation
Hiraoka et al. (31)	Apple Watch (PPG + ML); continuous post-surgery monitoring	Prospective observational	56	Post-cardiac surgery patients/67.1 years	Telemetry ECG	AUC 0.96 vs. telemetry ECG; sensitivity 92%, specificity 93%	Postoperative AF monitoring
Hibino et al. (32)	Wearable patch ECG (Carnation Monitor); 30-day post-discharge monitoring	Retrospective observational	144	Post-cardiac surgery patients at discharge/64.4 years	12-lead ECG review	POAF was detected in 17.4% post-discharge; 80% of events occurred ≥5 days after surgery	Postoperative AF monitoring

AF, atrial fibrillation; AHRE, atrial high-rate episode; CHA_2_DS_2_-VASc, congestive heart failure, hypertension, age ≥75 years (doubled), diabetes, stroke/transient ischemic attack/thromboembolism (doubled), vascular disease, age 65–74 years, sex category; ECG, electrocardiography; HR, hazard ratio; ML, machine learning; POAF, postoperative atrial fibrillation; PPG, photoplethysmography; PPV, positive predictive value; RCT, randomized controlled trial.

For active ECG screening, the population-based STROKESTOP trial demonstrated that intermittent screening among older adults (aged 75–76) significantly increased AF detection compared with routine care (23). Community pharmacy initiatives utilizing handheld ECG devices similarly proved feasible for high-risk cohorts (24). The REHEARSE-AF trial corroborated this, where twice-weekly screening via handheld AliveCor monitors yielded substantially higher AF detection rates than standard care (25). Expanding on intermittent strategies, the mSToPS trial revealed that immediate, continuous monitoring with wearable ECG patches in at-risk cohorts produced higher rates of AF diagnosis and subsequent anticoagulant initiation (26). Extended patch monitoring up to 14 days achieves excellent adherence (median analyzable time of 99%) and captures paroxysmal AF missed by traditional 48-hour windows (27). Furthermore, validation studies of integrated sensor textiles report signal fidelity comparable to standard Holter monitors (28). At a systemic level, platforms like China's “National ECG Network” leverage intelligent 12-lead recorders to facilitate real-time diagnostic interventions (29).

Recognizing wearables' capacity to identify occult AF missed by episodic methods, major clinical frameworks now formally endorse their use. Wearable-based AF screening holds a Class IIa recommendation in both the 2024 ESC (2) and the 2023 ACC/AHA/ACCP/HRS guidelines (1).

### 3.2 Monitoring of postoperative atrial fibrillation (POAF)

Postoperative atrial fibrillation (POAF) complicates 15%–40% of cardiovascular surgeries, with incidence surpassing 50% among octogenarians, substantially elevating the risk of embolic stroke, prolonged hospitalization, and mortality (30). Traditional bedside telemetry is restricted by patient mobility and abruptly discontinued upon discharge. Wearables, conversely, enable seamless rhythm surveillance across both inpatient and post-discharge recovery phases.

In the post-cardiac surgery setting, machine learning algorithms applied to Apple Watch PPG data accurately detected POAF against standard telemetry (31). For outpatient surveillance, wearable ECG patches effectively capture incident POAF within the critical 30-day post-discharge window (32). Similarly, smartphone-connected ECG devices in post-ablation patients maintain high diagnostic fidelity while reducing the frequency of in-person clinic and emergency department visits (33). By transcending geographical limitations, wearables reliably capture transient arrhythmic episodes and directly alleviate healthcare resource burdens.

### 3.3 Long-term home management

For patients with established AF, longitudinal rhythm monitoring is critical for evaluating treatment efficacy, guiding rate and rhythm control, and detecting post-ablation recurrence. The multicenter mAFA trial utilized PPG-enabled devices to provide real-time feedback on daily AF burden, facilitating timely clinical adjustments that significantly reduced cumulative AF burden and enhanced anticoagulation adherence (34). The iCARE-AF pilot study further explored intermittent anticoagulation guided by daily smartphone ECG transmissions, trending toward reduced bleeding events in low-risk paroxysmal AF patients (35).

By yielding continuous, quantifiable rhythm data, wearables are redefining AF from a categorical diagnosis to a continuously measurable physiological variable. However, dynamically tailoring anticoagulation based solely on device-detected AF burden mandates rigorous clinical outcome validation and highly individualized assessments of patient compliance and baseline stroke risk.

## 4 Challenges and future directions

### 4.1 Technical and regulatory challenges

Although pooled artificial intelligence sensitivities frequently achieve 90%–95% in controlled validation studies, real-world diagnostic accuracy remains inconsistent, varying considerably across clinical contexts and device architectures (36). PPG signals are particularly susceptible to environmental and physiological artifacts. Studies have reported degraded signal fidelity in individuals with darker skin pigmentation (37) or cutaneous conditions such as tattoos and scars. Motion artifacts and ambient light interference further obscure the distinction between true AF and physiological variations such as premature atrial or ventricular contractions (9, 38). While advanced signal processing algorithms attempt to mitigate these artifacts, they frequently result in substantial data loss (14).

Deployed at consumer scale, these technical imperfections magnify the absolute number of false-positive alerts, driving overdiagnosis and unnecessary therapeutic interventions that strain finite healthcare resources (20). The cost-effectiveness of population-wide wearable screening, therefore, remains unproven. The cascade of evaluations triggered by false positives—including unwarranted specialist consultations and supplementary testing—imposes a considerable financial burden on global healthcare systems (11).

Regulatory oversight presents a concurrent challenge. While agencies such as the US Food and Drug Administration apply stringent standards to medical-grade sensors, unified global standards for consumer-facing devices remain absent. Addressing this gap requires establishing standardized data formats, universal performance benchmarks, and rigorous validation protocols for AF detection. Future regulatory frameworks must mandate multicenter, multiethnic clinical trials to validate device accuracy across historically underrepresented populations, including the elderly, multi-morbid, and lower-income demographic groups.

Broader integration of wearables into established diagnostic and therapeutic pathways depends on achieving performance that consistently rivals clinical-grade ECG. Although major international guidelines now formally recognize wearable-based AF screening, their recommendations diverge in scope and strength. The 2024 ESC guidelines (2) assign a Class IIa recommendation (Level of Evidence B) for opportunistic screening using wearable or handheld ECG devices in individuals aged 65 and older. The 2023 ACC/AHA guidelines (1), by contrast, withhold broad population-wide endorsement, emphasizing individualized decision-making based on AF episode duration and baseline stroke risk. Contemporary Chinese guidelines (3, 4) similarly acknowledge the diagnostic potential of wearable technologies but note that robust evidence from large-scale, multicenter, domestic populations remains limited. Harmonizing these divergent frameworks will require dedicated pragmatic outcome trials across diverse healthcare systems. Bridging this evidence gap requires implementation science strategies that prospectively evaluate not only diagnostic accuracy but also workflow integration and long-term patient outcomes in real-world settings.

### 4.2 Data silos and EHR interoperability

Beyond diagnostic accuracy, the clinical utility of wearable sensors is substantially constrained by fragmented data ecosystems. Massive volumes of physiological data currently remain confined to proprietary smartphone applications, generating isolated data silos. Without standardized data exchange protocols—such as HL7 Fast Healthcare Interoperability Resources (FHIR)—or direct integration into electronic health record (EHR) pipelines, this lack of semantic interoperability places a considerable burden on healthcare providers. Clinicians are frequently required to manually review raw, unstructured data during time-constrained encounters, a process that risks information overload rather than generating actionable clinical insights (15).

Addressing this barrier requires future digital health infrastructures to prioritize systemic interoperability through standardized data exchange protocols, including FHIR (39). The development of intelligent, clinician-facing AI dashboards is equally important—interfaces capable of distilling months of raw sensor data into concise rhythm burden summaries embedded directly within existing EHR workflows. Establishing secure, automated, and standardized data pipelines represents a critical step in transitioning wearable sensors from isolated consumer accessories into integrated clinical tools.

### 4.3 Physical safety and data security

Beyond diagnostic accuracy and interoperability, the physical safety of wearable hardware warrants careful clinical consideration. Emerging reports suggest that magnetic components embedded in certain smartwatch wristbands may induce electromagnetic interference with implantable cardiac devices, including pacemakers and implantable cardioverter-defibrillators (ICDs) (40). Continuous skin-contact sensors frequently incorporate acrylic adhesives and metallic alloys, which have been occasionally implicated in contact dermatitis and localized allergic reactions (41). Mitigating these risks requires manufacturers to adopt biocompatible, hypoallergenic materials and optimize sensor architecture to minimize electromagnetic interference. Robust post-market surveillance is also necessary to evaluate the physical safety profile of these devices during prolonged use.

The exponential accumulation of continuous physiological data introduces concurrent cybersecurity vulnerabilities. Transmitting health metrics from consumer wearables to cloud platforms necessitates rigorous encryption standards to prevent data breaches and unauthorized exploitation. Compliance with established regulatory frameworks—including the Health Insurance Portability and Accountability Act (HIPAA) in the United States and the General Data Protection Regulation (GDPR) in the European Union—provides an essential foundation for data protection. Transparent communication with patients about data ownership, storage protocols, and third-party sharing is equally important for preserving public trust and supporting sustained adherence to remote monitoring programs.

### 4.4 Evidence gaps, digital health equity, and usability

A fundamental limitation in current wearable research is the demographic discrepancy between study cohorts and the target clinical population. Performance data among elderly, multi-morbid patients—who represent the primary demographic at risk for AF—remain scarce. Many large-scale trials feature cohorts skewed toward younger, healthier, and more technologically literate individuals, reflecting the general consumer base of smart electronics. This discrepancy leaves a notable gap in the evidence base for higher-risk older adults and those with multiple comorbidities (20). This demographic imbalance also raises concerns for digital health equity. The predominant adoption of these devices among affluent populations risks exacerbating existing healthcare inequalities, potentially marginalizing socioeconomically disadvantaged groups who bear a disproportionate burden of AF-related stroke. Ensuring equitable access and user-friendly interfaces is therefore important to prevent further widening of the digital divide.

Beyond demographic disparities, a substantial proportion of existing studies feature short follow-up durations and lack evidence regarding the long-term impact of wearables on hard clinical outcomes, such as stroke reduction or mortality. The implementation of these technologies across diverse clinical settings also remains incompletely characterized.

Future implementation strategies should prioritize large-scale, prospective outcome trials designed to assess not only AF detection rates but also clinical utility, healthcare utilization, and long-term stroke prevention efficacy. The cost-effectiveness of population-wide wearable AF screening remains uncertain, as the potential economic benefits of earlier stroke prevention must be carefully weighed against the downstream costs of false-positive evaluations and increased healthcare utilization (11, 26).

Usability barriers—including complex interfaces, limited physical comfort, insufficient battery life, and prohibitive device costs—have been widely reported in real-world observational studies and represent a significant impediment to long-term adherence among older adults (42, 43). Addressing these limitations requires user-centric design approaches tailored for this population, incorporating larger interface elements, simplified navigation, extended battery life, voice-activated controls, and accessible application feedback. Such design optimizations may improve long-term adherence and support the broader integration of wearable devices into AF management pathways (42, 43).

### 4.5 The clinical dilemma: subclinical AF and the anticoagulation threshold

The widespread adoption of consumer wearables has introduced a profound clinical dilemma surrounding the detection and management of subclinical atrial fibrillation (SCAF), a challenge brought into sharp focus by the contrasting findings of the NOAH-AFNET 6 and ARTESiA trials (44, 45). In contemporary clinical nomenclature, device-detected AF is broadly termed SCAF; when captured by continuous implantable cardiac devices, it is formally defined as atrial high-rate episodes (AHRE) (1, 2). Unlike clinical AF documented by standard 12-lead ECG, wearable-detected SCAF is frequently asymptomatic and characterized by markedly shorter episode durations (1). The sheer scale of consumer wearable adoption has led to a substantial increase in SCAF detection (20, 26), confronting clinicians with a pressing question: do these asymptomatic patients derive net clinical benefit from the initiation of oral anticoagulants (OACs)?

Historically, OAC initiation was guided primarily by clinical risk scores such as the CHA_2_DS_2_-VASc score, often independent of AF burden. Two landmark randomized controlled trials—NOAH-AFNET 6 and ARTESiA—have since provided critical, and seemingly contradictory, evidence bearing on this question. A detailed comparison of their designs and findings is essential to contextualize their implications for wearable-detected AF (Table 3).

**Table 3:** Comparison of NOAH-AFNET 6 and ARTESiA trials and their implications for wearable-detected AF.

Characteristic	NOAH-AFNET 6 (44)	ARTESiA (45)	Implications for wearable-detected AF
Population	Age ≥65 years, CHA_2_DS_2_-VASc ≥2	CHA_2_DS_2_-VASc ≥3	Wearable users are often younger/lower risk; trial data may overestimate benefit in typical wearable cohorts.
AHRE/SCAF definition	AHRE ≥170 bpm, ≥6 min	SCAF ≥6 min to ≤24 h	Wearable-detected episodes are typically shorter and unconfirmed; confirmation by ECG is mandatory.
Detection device	Pacemakers, ICDs, ILRs	Pacemakers, ICDs, ILRs	Implantable devices provide continuous, high-fidelity data; wearables are intermittent and prone to artifacts.
Sample size	2,536	4,012	Both were adequately powered for their respective endpoints
Intervention	Edoxaban vs. Placebo	Apixaban vs. Aspirin	Choice of OAC and comparator may influence risk-benefit assessment
Primary endpoint	CV death, stroke, systemic embolism	Stroke or systemic embolism	Stroke reduction must be weighed against bleeding in lower-risk populations.
Stroke reduction	HR 0.81 (95% CI 0.60–1.08), *p* = 0.15 (NS) (44)	HR 0.63 (95% CI 0.45–0.88), *p* = 0.007 (45)	Benefit likely restricted to higher-risk patients (CHA_2_DS_2_-VASc ≥3)
Major bleeding	HR 2.10 (95% CI 1.30–3.38), *p* = 0.002 (44)	HR 1.80 (95% CI 1.26–2.57), *p* = 0.001 (45)	Bleeding risk consistently increased; HAS-BLED assessment essential
Guideline implication	OAC is not routinely recommended for low-burden AHRE	OAC may be considered for SCAF with CHA_2_DS_2_-VASc ≥3	Wearable alerts require ECG confirmation before applying these trial findings

AHRE, atrial high-rate episode; CHA_2_DS_2_-VASc, congestive heart failure, hypertension, age ≥75 years, diabetes, stroke/transient ischemic attack, vascular disease, age 65–74 years, sex category; CV, cardiovascular; ECG, electrocardiography; HR, hazard ratio; ICD, implantable cardioverter-defibrillator; ILR, implantable loop recorder; NS, not significant; OAC, oral anticoagulant; SCAF, subclinical atrial fibrillation; TIA, transient ischemic attack. A detailed discussion of the differences between implantable device-detected and wearable-detected AF is provided in Section 4.5.

NOAH-AFNET 6 enrolled 2,536 patients aged 65 years or older with a CHA_2_DS_2_-VASc score of 2 or higher and device-detected AHRE (≥170 bpm for ≥6 min) identified by pacemakers, defibrillators, or implantable loop recorders (44). Edoxaban did not significantly reduce the composite outcome of cardiovascular death, stroke, or systemic embolism compared with placebo (HR 0.81, 95% CI 0.60–1.08, *p* = 0.15), while significantly increasing major bleeding (HR 2.10, 95% CI 1.30–3.38, *p* = 0.002) (44). ARTESiA enrolled 4,012 patients with SCAF lasting between 6 min and 24 h and a CHA_2_DS_2_-VASc score of 3 or higher (45). Apixaban significantly reduced stroke or systemic embolism compared with aspirin (HR 0.63, 95% CI 0.45–0.88, *p* = 0.007), while also significantly increasing major bleeding (HR 1.80, 95% CI 1.26–2.57, *p* = 0.001), yielding an absolute stroke reduction of 0.6% per year offset by an absolute bleeding increase of 0.8% per year (45).

The divergent conclusions of these trials can be reconciled by examining key differences in their study designs and patient populations. First, ARTESiA enrolled a higher-risk cohort (CHA_2_DS_2_-VASc ≥3 vs. ≥2 in NOAH-AFNET 6), yielding greater absolute benefit from anticoagulation. Second, the comparator agents differed—placebo in NOAH-AFNET 6 vs. aspirin in ARTESiA. Third, the specific SCAF and AHRE episode duration thresholds differed; while both trials required a minimum duration of 6 min, ARTESiA specifically excluded episodes exceeding 24 h. These distinctions reflect a fundamental principle: SCAF and AHRE do not constitute a monolithic entity. The net clinical benefit of OAC depends on the interplay between baseline stroke risk, bleeding risk, and AF burden characteristics. In the lower-risk NOAH-AFNET 6 population, the absolute stroke rate in the placebo arm was only 1.1% per year, and the bleeding risk associated with edoxaban outweighed the non-significant stroke reduction. In the ARTESiA cohort, a higher baseline stroke risk enabled apixaban to yield a 37% relative risk reduction in stroke—a net benefit that many patients and clinicians may consider acceptable despite the concurrent increase in bleeding.

Critically, both trials enrolled patients whose AHRE or SCAF were detected exclusively by implantable cardiac devices, which provide continuous, high-fidelity intracardiac electrogram monitoring over years. This differs fundamentally from consumer wearable detection in three respects. First, implantable devices seamlessly capture every episode exceeding a programmed duration threshold over months to years of uninterrupted monitoring, whereas wearables provide only intermittent snapshots—such as 30-second ECG recordings, or short-term continuous data lasting 14–30 days for patch monitors. PPG-based smartwatches monitor only during active wear time and are frequently removed during sleep; consequently, wearable-detected AF episodes represent an unknown fraction of the true underlying AF burden. Second, the diagnostic specificity of intracardiac electrograms for AF approaches 100%, whereas PPG-based algorithms report positive predictive values of 84%–98% in controlled screening studies (20–22, 36), with accuracy likely lower in real-world settings where motion artifacts, ectopic beats, and poor signal quality are more prevalent. Third, trial participants were predominantly older adults (mean age 72–78 years) with elevated stroke risk, whereas large-scale wearable screening studies have typically enrolled substantially younger cohorts (mean age 35–47 years) (20–22). Given these differences in monitoring intensity, diagnostic accuracy, and population risk profile, the risk-benefit estimates from NOAH-AFNET 6 and ARTESiA cannot be directly extrapolated to wearable-detected AF. Dedicated prospective outcome trials are therefore needed to evaluate OAC efficacy and safety specifically in patients identified through consumer wearable devices.

Both the 2023 ACC/AHA/ACCP/HRS guidelines (1) and the 2024 ESC guidelines (2) recommend that OAC decisions be individualized based on AF episode duration, CHA_2_DS_2_-VASc score, and bleeding risk. Episodes lasting less than 5 min generally do not warrant OAC therapy. Episodes between 5 min and 24 h may merit OAC in patients with elevated stroke risk, with careful weighing of bleeding risk. Episodes exceeding 24 h should be managed similarly to clinical AF. For wearable-detected AF, diagnostic confirmation via physician-interpreted ECG remains an absolute prerequisite before any therapeutic decision. Building upon these data, we propose a structured clinical approach: PPG-based alerts should be confirmed with single-lead or 12-lead ECG before clinical action; thromboembolic and bleeding risks should be assessed using CHA_2_DS_2_-VASc and HAS-BLED (46); AF burden should be quantified through continuous ECG monitoring rather than intermittent PPG spot-checks; and OAC decisions should be individualized—conservative management for episodes under 5 min, guideline-directed OAC for episodes exceeding 24 h, and shared decision-making for intermediate episodes between 5 min and 24 h, balancing stroke and bleeding risk alongside patient preferences.

Although the preceding discussion has focused on the specific dilemma of anticoagulation for SCAF, several broader limitations of the current evidence base warrant explicit acknowledgment. A major future direction for wearable technologies is to move beyond binary AF-vs.-non-AF alerts toward the accurate, continuous quantification of AF burden, thereby distinguishing sustained pathological episodes from brief, isolated anomalies. Only by providing such granular, actionable physiological data can wearables truly assist clinicians in navigating this complex antithrombotic dilemma.

## 5 Limitations

This narrative review is inherently susceptible to selection bias. Unlike systematic reviews governed by rigid retrieval protocols, our framework relies on the prioritized inclusion of literature, which may influence the subjective interpretation of evidence. The rapid iteration of wearable hardware and proprietary AI algorithms presents another limitation; diagnostic metrics from earlier trials may fail to reflect contemporary software capabilities, as manufacturers continuously refine diagnostic algorithms post-launch to alter real-world accuracy.

Furthermore, the current evidence base is disproportionately derived from affluent, technologically literate cohorts. Compounding this demographic imbalance is the high prevalence of manufacturer-sponsored validation studies, introducing potential publication bias. Independent, real-world data validating wearable efficacy specifically in elderly and multi-morbid patients—the primary populations at risk for AF—remain critically deficient.

Robust evidence regarding the cost-effectiveness of wearable-based AF management is also sparse. Downstream healthcare expenditures triggered by false-positive evaluations risk offsetting the economic benefits of earlier detection. Addressing this specific evidence gap is critical to justifying the widespread adoption of these technologies, particularly within resource-limited healthcare settings. Finally, while our literature search spanned English and Chinese databases to capture global and regional perspectives, restricting inclusion to peer-reviewed publications inherently excludes grey literature, such as manufacturer registries and medical device white papers, which may contain supplementary safety and performance metrics.

## 6 Conclusion

Wearable devices have emerged as valuable instruments for AF identification and management, enabling a shift from episodic clinical assessment to continuous, personalized digital monitoring. Advances in flexible electronics and fabric-based sensors continue to broaden the scope of remote physiological data acquisition, as illustrated by multimodal devices such as smart belts capable of measuring impedance and ECG (47). Realizing the full clinical potential of these technologies, however, requires moving beyond arrhythmia detection to address the complex management of SCAF (44, 45).

Several priorities will shape future progress. AI architectures must be refined to accurately quantify daily AF burden, reducing false-positive alerts and the downstream consequences of overdiagnosis and unwarranted anticoagulation. Standardized data exchange pipelines—such as FHIR—are needed to dismantle proprietary data silos and integrate wearable metrics directly into hospital EHR workflows. Concurrently, hardware and application design must prioritize age-appropriate, user-centric models to resolve usability barriers for elderly and multi-morbid populations. The convergence of 5G telecommunications, artificial intelligence, and the Internet of Medical Things (IoMT) positions AF management to evolve into an interconnected ecosystem of precision early warning, personalized intervention, and equitable healthcare access. Transitioning wearables from isolated consumer electronics to fully integrated diagnostic tools could contribute to reducing the global cardiovascular burden. This goal will require sustained interdisciplinary collaboration among engineers, clinicians, regulatory bodies, and patients—ensuring that wearable devices deliver measurable and equitable improvements in cardiovascular outcomes.
